# Postural Instability in Subjects With Usher Syndrome

**DOI:** 10.3389/fneur.2019.00830

**Published:** 2019-08-08

**Authors:** Simona Caldani, Maria Pia Bucci, Maud Tisné, Isabelle Audo, Thierry Van Den Abbeele, Sylvette Wiener-Vacher

**Affiliations:** ^1^UMR 1141 Inserm, Robert Debré Hospital, Université de Paris, Paris, France; ^2^FEE, ENT Department, Center for Children Balance Disorders Evaluation, Robert Debré Hospital, Paris, France; ^3^CHNO des Quinze-Vingts, DHU Sight Restore, INSERM-DHOS, Paris, France

**Keywords:** Usher syndrome, postural control, visuo-vestibular inputs, proprioception, rehabilitation

## Abstract

This study investigated postural performances and vestibular impairment in Usher patients. The three groups studied were: 11 patients with Usher type I (with visual and vestibular impairment), 14 patients with Usher type II (with only visual impairment), and 14 healthy control subjects. Postural stability was measured with a Framiral Multitest Equilibre platform with three visual conditions: eyes open (EO), eyes closed (EC), and vision disturbed by optokinetic stimulation (OPT), and two different postural conditions: stable or unstable platform. The surface and mean velocity of the center of pressure displacement (CoP) were measured and a postural instability index (PII) was calculated. Usher type I and II patients were more unstable than control subjects, but only for the unstable platform. Patients with Usher type I (with severe vestibular impairment) were also significantly more unstable than patients with Usher type II (with normal vestibular function) on the unstable platform. The severity of the vestibular impairment was correlated with the surface of the CoP displacement. We suggest that poor postural control of Usher patients is due to the abnormalities in their visual and, when defective, vestibular inputs. Measurements of postural stability on an unstable platform can distinguish type I from type II Usher patients. We emphasize the importance of multisensory evaluation in these patients to guide development of personalized visuo-vestibular rehabilitation techniques to improve their postural stability and improve their quality of life.

## Highlights

- Patients with Usher (type I and II) are more unstable with respect to controls on unstable platform only.
- Patients with Usher type I are significantly more unstable than patients with Usher type II and controls particularly with eyes opened.
- The postural imbalance, observed in patients with Usher type I, is correlated to the severity of their vestibular impairment.
- The absence of or abnormal visual and/or vestibular inputs is probably the cause of such a poor postural control in patients with Usher syndrome.
- The parameter of the surface of the center of pressure on unstable platform could be used to differentiate the behavior of patients with Usher type I and patients with Usher type II.
- In the future, the parameter of the surface could also be used to evaluate the effect of postural reeducation as well as the rate of improvement in the quality of life for these patients.

## Introduction

Maintaining stable posture is necessary for performing many daily tasks (1). Postural control corresponds to a complex neurological function that relies on sensory inputs that are conveyed by the visual, proprioceptive, and vestibular systems (2). A deficit in one of these inputs can lead to postural instability [e.g., Bucci et al. (3)].

Usher syndrome results from a genetic disorder and is characterized by the association of sensorineural hearing loss, retinitis pigmentosa (RP), and in some cases, vestibular dysfunction (4). The RP consists of a degenerative genetic disease of the retina leading to a gradual loss of peripheral vision and, ultimately, blindness. In the literature there are three different types of Usher syndrome: type I, with profound hearing loss, congenital vestibular dysfunction, and different degrees of RP, is diagnosed during the first decade of life. Type II Usher syndrome is characterized by hearing loss, normal vestibular function, and RP of varying degree. This is often diagnosed during the second or third decade of life. Usher type III, with progressive deafness and/or vestibular disorders, is usually diagnosed in the first decade of life (5). These patients face progressive multisensory handicaps that lead to loss of balance control and motor independence.

To our knowledge, only one study explored postural control in patients with Usher syndrome. In 1991, Pyykko et al. (6) compared postural stability on a stable platform, with or without destabilizing foam pads, in 10 blind subjects and 10 patients with Usher syndrome. They found that the Usher syndrome patients were more unstable while standing on foam pads but not on solid ground. They suggested that Usher patients could compensate for their vestibular deficit but were not able to compensate for their poor visual input. However, in this study the type of Usher syndrome was not specified, nor the degree of retinal or vestibular impairment.

Perturbations of visual information and vestibular information are known to have an impact on postural stability and can be more or less compensated with central sensory reweighting processes. For example, postural stability is significantly impaired in children with strabismus whose vision is perturbed relative to non-strabismic children (7–9). Bucci et al. (10) showed that the use of foam pads of different thicknesses could have different effects on postural stability in strabismic children. Thick foam pads (15 cm) significantly perturbed postural stability compared to controls, while thin foam pads (4 mm thick) significantly improved postural stability. The authors suggested that thin foam pads slightly increase proprioceptive stimulation in these visually deficient children where proprioceptive abilities are particularly well-developed for compensation.

Postural instability in age-related macular degeneration (AMD) patients (a main cause of blindness and visual deficits in adults) has also been the subject of several publications. Kotecha et al. (11) compared the effect of foam pads on the postural stability of elderly AMD patients and age-matched healthy subjects. They found that AMD patients were more unstable than the controls in both postural conditions (with or without foam pads), suggesting that elderly subjects may not be able to reweight different sensory inputs to maintain their stability. More recently, Chatard et al. (12) studied the impact of AMD on postural sway and observed that AMD subjects were more unstable than controls, again interpreted as being most likely due to the absence of postural adaptive mechanisms which could compensate for their visual deficits.

This is consistent with a study by Assländer and Peterka (13) which showed that healthy adult subjects were able to reweight different sensory information in order to maintain their stability. Peterka (14) compared postural stability during standing in four patients with bilateral vestibular loss (45–58 years old) and in eight control subjects (24–46 years old) during unpredictable rotation of the visual surround and/or support surface. He found that when both visual and proprioceptive inputs were available, the majority of patients (3/4) were able to maintain postural stability as well as control subjects. This supports Carver et al.'s (15) suggestion that adaptative mechanisms can permit compensation when one sensory input is defective by reweighting the integration of other sensory inputs.

The vestibular system also contributes to maintain an internal representation of the body position and movement in space. Several studies found that patients with a vestibular loss had difficulties with spatial orientation and environmental exploration (16–18). In the Equitest, vestibular deficit is characterized by a fall or severe instability when visual inputs are suppressed (eye closed or stabilized vision) and proprioceptive information is simultaneously perturbed (horizontal translation of the feet support) (19). It is known also that this postural instability due to vestibular impairment can improve with the compensation processes, particularly with partial vestibular loss and physical therapy, but never completely in case of complete bilateral vestibular loss (20).

In Usher syndrome, hearing loss is part of the multisensory deficit. It can be of various degrees that require hearing aids or cochlear implantation. It is known that auditory information plays a role in spatial representation and, to some extent, in postural stability, and this was shown in patients with hearing loss (21). In a recent study, Mazaheryazdi et al. (22) explored postural function in 25 children (mean age: 9.3 years) with bilateral vestibular hypofunction deficit and unilateral or bilateral cochlear implants (CI) in two conditions: cochlear implant turned “on” and “off.” The authors observed that auditory information (with CI on) could improve postural stability and reduce body sways, suggesting that loss of hearing could lead to postural perturbations. Shayman et al. (23) showed in adult subjects (23–84 years old) with cochlear implants that turning on cochlear implants bilaterally improved head and trunk stability.

The aim of the present study was to measure postural performance in patients with Usher type I vs. Usher type II in different challenging visual and proprioceptive conditions in order to better evaluate the impact of vestibular and visual function on postural control in these patients.

Our hypothesis was that patients with Usher type I would be more unstable when compared to patients with Usher type II, particularly on the unstable platform (where proprioceptive inputs are perturbed), depending on the presence of vestibular impairment associated to the visual impairments.

## Materials and Methods

### Participants

#### Clinical Characteristics

Two groups of patients with Usher syndrome (genetically identified for their type) were recruited in this study. The first group was composed of 11 patients with Usher type I (mean age: 27.1 ± 3.2 years), and the second of 14 patients with Usher type II (mean age: 42.9 ± 3.2 years). A third group of 14 healthy control (CTR) subjects (mean age: 28.26 ± 0.83 years) was also included. Note that the Usher type I patients were younger than the Usher type II because in Usher II the visual impairment starts later and can delay the diagnosis to older ages.

### Population

Control subjects were recruited in Robert Debré Hospital (members of the staff, siblings of patients…). Exclusion criteria were: any known neurological disorders, visual impairment, any known vestibular disorder, and known orthopedic disorder or surgeries.

Patients with Usher (type I, II) had a complete vestibular examination (semicircular canal and otolith systems) by the same specialist. All subjects underwent a full vestibular evaluation including head impulse test, caloric test, EVAR (Earth Vertical Axis Rotation (EVAR) tests for canal function, OVAR (Off Vertical Axis Rotation) test, and good conduction VEMP (Vestibular-Evoked Myogenic Potential) for otholith function assessment, as well as a neurological and audiological evaluation (24, 25). Exclusion criteria for Usher (type I, II) were: the lack of molecular diagnosis of Usher's syndrome. Only six patients of the Usher I group (but none in Usher 2) retained some remaining vestibular canal function at high and low head velocities and no otolith function.

We divided the population into three subgroups according to the degree of vestibular impairment [similar to Strupp et al. (26)]: level 1 means complete vestibular areflexia (CVA), level 2 means a partial vestibular deficit (PVD) and level 3 means normal vestibular function (NVF). Note that here CVA corresponds to an absence of vestibulo-ocular reflex (VOR) in response to rotatory head stimulation (a VOR gain at the vHIT <0.05 for the three canals on both sides for a head angular velocity >150°/s), no caloric-induced nystagmus for stimulation with 20°C cold water on each side, and a horizontal angular VOR gain <0.1 upon sinusoidal stimulation on a rotatory chair (0.05 Hz, V_max_ = 25°/s). PVD was defined by responses lower than the normal values at one of the three tests: vHIT, caloric test, and sinusoidal stimulation on a rotatory chair. Response to vHIT was considered lower than normal when the VOR gain was <0.80 for one or more of the six canals (for a head angular velocity >150°/s). Response to caloric test was considered lower than normal when the reflectivity (calculated from the quick phase frequency for 30 and 44°C) was <1 Hz, or when response was obtained only with 20°C cold water (and not for 30 and 44°C) on one or both sides. Response to the sinusoidal stimulation on a rotatory chair was considered lower than normal when the VOR gain was <0.30. Only six patients with Usher I had remaining vestibular function: 4/6 had gain at the vHIT from 0.53 to 0.23 (in one case for one anterior canal, in 3/4 patients for both anterior canals, in 1/4 patients for both posterior canals). All six patients had responses to caloric test (1/6 had no response to caloric test, 3/6 had partial response with reflectivity >1 Hz, 2/6 with normal reflectivity of 1.5 Hz in one side). None of the six patients had response to horizontal angular rotatory test.

All patients had an ophthalmological evaluation of their binocular eye field. Clinical data of patients are presented in Tables 1A,B. Note that for patients with Usher type I the age range was 17–54 years old, and for patients with Usher type II it was 17–60 years old. The binocular visual field of all patients had an ophthalmological evaluation with the Goldmann perimetry test. Binocular visual field charts were used to quantify the degree of visual deficit. They are divided by radial lines (separated by 15°) and concentric lines (separated by 10°). They appear as a dart board with 400 compartments. For each patient we counted the number of compartments that were surrounded by the zone of visual sensitivity (isopters) of the patient for the central and the peripheral visual fields. The results were expressed as a percentage of the entire visual binocular field. On the chart, the peripheral visual field is defined as the area of vision in an annulus extending from 10 to 90° radii. The central visual field was defined as extending from the center to a circle of radius 10°. The effective visual field was expressed as a percentage of the entire field. In order to study the impact of the vestibular deficit of the Usher' patients, we used the Dizziness Handicap Inventory (DHI) questionnaire scoring from Jacobson and Newman (27) (see Tables 1A,B).

**Table 1:** Clinical characteristics of patients with Usher type I **(A)** and Usher type II **(B)**.

**Vestibular screening**	**DHI**	**Peripheral visual field (PVF)**	**Champ visual central (CVF)**	**Auditory rehabilitation**
**(A)**				
PVD	34	12	100	Hearing aid
CVA	34	1,56	100	Cochlear implant
CVA	6	78,64	100	Cochlear implant
PVD	26	100	100	Cochlear implant
CVA	26	0,52	100	Hearing aid
PVD	14	25	100	Hearing aid
CVA	10	100	100	Cochlear implant
PVD	42	7,29	100	Hearing aid
CVA	80	2,02	100	Hearing aid
PVD	34	10,94	100	Hearing aid
PVD	18	78,12	100	Hearing aid
**(B)**				
NVF	8	45,83	100	Hearing aid
NVF	16	71,09	100	Hearing aid
NVF	26	0	50	Hearing aid
NVF	14	0	50	Hearing aid
NVF	4	16,93	100	Hearing aid
NVF	12	71,61	100	Hearing aid
NVF	6	0	100	Hearing aid
NVF	0	27,06	75	Cochlear implant
NVF	2	5,73	100	Hearing aid
NVF	26	0	25	Hearing aid
NVF	0	75	100	Hearing aid

*CVA means complete bilateral vestibular areflexia; PVD, a partial vestibular deficit; NVF, a normal vestibular function; DHI, Dizziness Handicap Inventory score; PVF, the peripheral area of the visual field; CVF, the area of the central visual field. In the last column is indicated the type of auditory rehabilitation*.

The investigation adhered to the principles of the Declaration of Helsinki and was approved by our Institutional Human Experimentation Committee (Comité de Protection des Personnes CPP, Ile de France). After the procedure had been explained, written informed consent was obtained from the participants and their parents when the participants were under the age of 16.

### Postural Evaluation

Patients had to stand upright on the Framiral^®^ platform with their arms along the side of the body and their feet on the center of the platform. The surface of the center of pressure displacement (CoP) was measured with stable and unstable platform under three different viewing conditions: eyes open (EO), fixating a target at a distance of 250 cm (target projected on a screen in front of the subjects at their eye level), eyes closed (EC), and vision disturbed by optokinetic stimulation (OPT) from an optokinetic ball. The platform could be moved by 62 mm in the anteroposterior and mediolateral directions. Oscillations allow forward and backward translations, with a constant linear velocity which may vary from 0.03 to 0.07 m/s with a frequency of 0.25 Hz [for details, see Goulème et al. (28)].

The OPT condition was obtained by a planetarium projection on a wall at a distance of 250 cm from the subject and rotated with a mean velocity of 15°/s, randomly from top to bottom and from bottom to top. The displacement of the CoP was sampled at 40 Hz and digitized with 16-bit precision. Each postural recording was performed for 30 s. Postural and visual tests were randomly tested. These conditions were used to better evaluate the use of both somatosensory and/or vestibular input since vision is altered. For patients with cochlear implant (*n* = 5) the test was done two times with cochlear implant on than off. For these five patients the data obtained with the cochlear implant on have been used for the study.

### Data Analysis

Postural performance was evaluated by measuring the surface of the CoP (in cm^2^), the mean velocity of the CoP (mm/s), and the calculated postural instability index (PII). The surface of the CoP corresponded to the area of an ellipse encompassing 90% of all CoP data point excursions. The mean velocity of CoP is an index of the neuromuscular activity required to achieve postural control (29, 30). The PII quantified the postural stability by using the spectral power index (PI) and the canceling time (CT) for both medio-lateral (x) and antero-posterior (y) directions. It was calculated as follows: PII = Σx Σy PI (F1, F2, F3)/CT (F1, F2, F3), where PI and CT are the spectral power index and cancellation time for each of the three frequency bands (low, from 0.05 to 0.5 Hz: F1; medium, from 0.5 to 1.5 Hz: F2 and high, higher than 1.5 Hz: F3). Higher values of PII indicate a poor postural stability (3).

### Statistical Analyses

In order to compare the different groups of subjects (Usher type I, Usher type II, and CTR) in two postural (platform stable and unstable) and three visual conditions (EO, EC, and OPT) and in order to control the age-related effects on postural stability, analysis of covariance (ANCOVA) on postural parameters with participants' age as a covariable was performed. If significant effects were found, *post-hoc* Bonferroni was performed. Moreover, analysis of variance (ANOVA) was also made in order to compare the different groups of patients (Usher type I and II) on the DHI score.

Correlations between the vestibular function, as well as visual deficit and the surface area of CoP under unstable platform with EO condition, have been examined in the two groups of patients tested using Spearman's correlation coefficients.

The effect of a factor was considered significant when the *p*-value was below 0.05.

## Results

### Surface of the CoP

**Figure 1:**
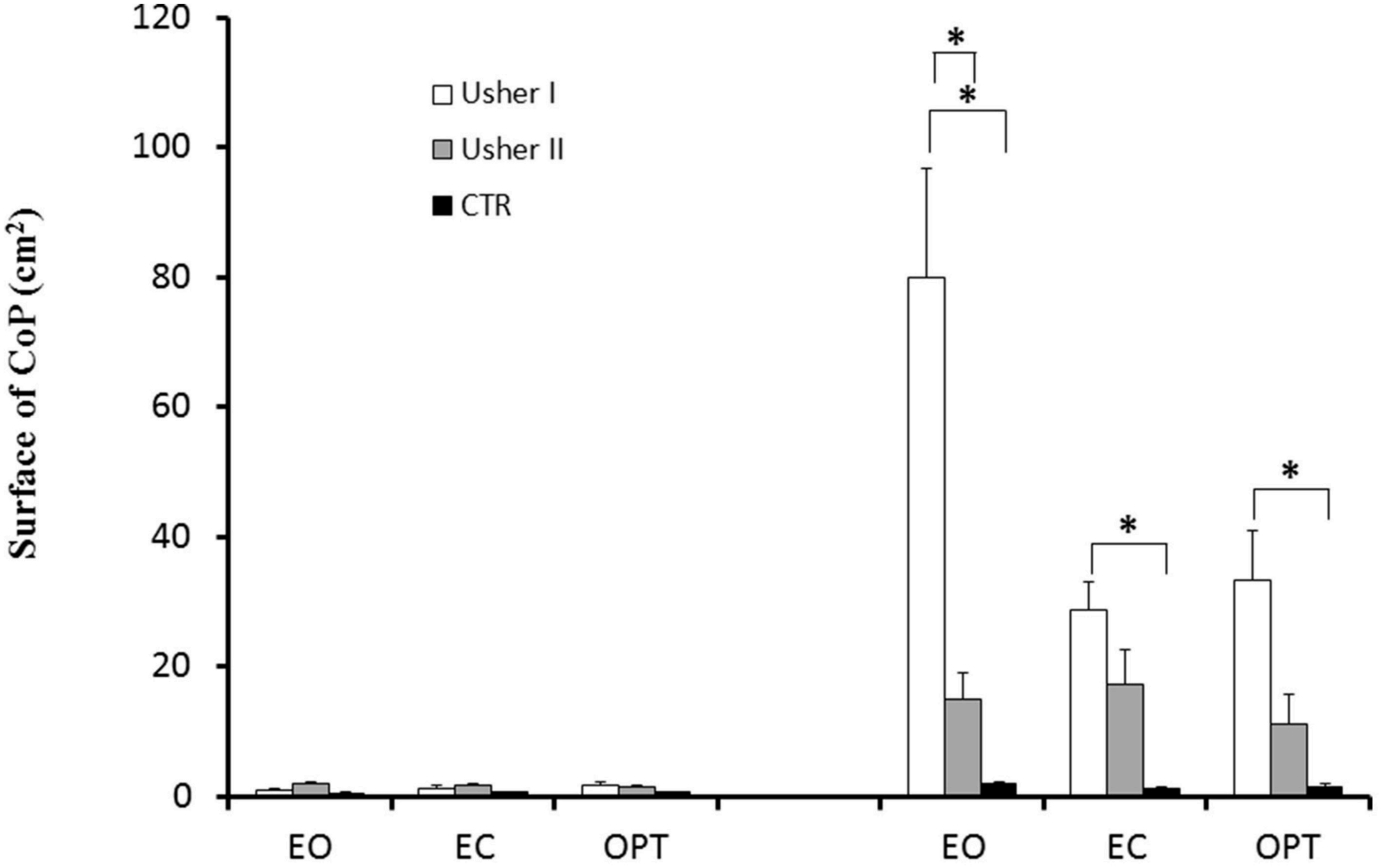
Means and standard deviations of surface of the CoP (cm^2^) in two different postural conditions (standing on stable and unstable platform) for the three visual conditions tested in patients with Usher type I and type II and in controls. Asterisks indicate significant difference between the groups (ANCOVA-Bonferroni).

In Figure 1, the surface of the CoP displacement recorded under different postural and visual conditions are reported for the three groups of subjects; ANCOVA showed a significant group effect [*F*_(2, 38)_ = 21.53; *p* < 0.0001]. Bonferroni test showed that only patients with Usher type I had a larger surface of CoP when compared to CTR and Usher type II (both *p* < 0.0001), with no differences between Usher type II and CTR. A postural condition effect was also found [*F*_(1, 38)_ = 9.97; *p* < 0.003], showing that surface area of CoP was significantly larger on the unstable platform.

Analysis of covariance (ANCOVA) also found a significant interaction between Posture and Group [*F*_(2, 38)_ = 22.82; *p* < 0.0001]; Bonferroni test showed that Usher type I had a larger surface of CoP only on unstable platform when compared to CTR and to Usher type II (both *p* < 0.0001). Moreover, Usher type II had a larger surface of CoP when compared to CTR (*p* < 0.02).

Analysis of covariance (ANCOVA) reported a significant interaction between Vision and Group [*F*_(4, 76)_ = 4.04; *p* < 0.005]; Bonferroni test showed that patients with Usher type I had a larger surface during only EO and OPT when compared to CTR (respectively, *p* < 0.0001 and *p* < 0.02), and only during EO condition when compared to Usher type II (*p* < 0.0001). In contrast, there was no difference between CTR and Usher type II.

Finally, ANCOVA showed a significant interaction between Group, Vision, and Posture [*F*_(4, 76)_ = 4.21; *p* < 0.004]. On unstable platform, Bonferroni test showed that patients with Usher type I had a larger surface of CoP in all visual conditions compared to CTR (respectively, *p* < 0.0001, *p* < 0.03, *p* < 0.001), and only during EO condition when compared to patients with Usher type II (*p* < 0.0001). There was not any difference between Usher type II and CTR.

**Figure 2:**
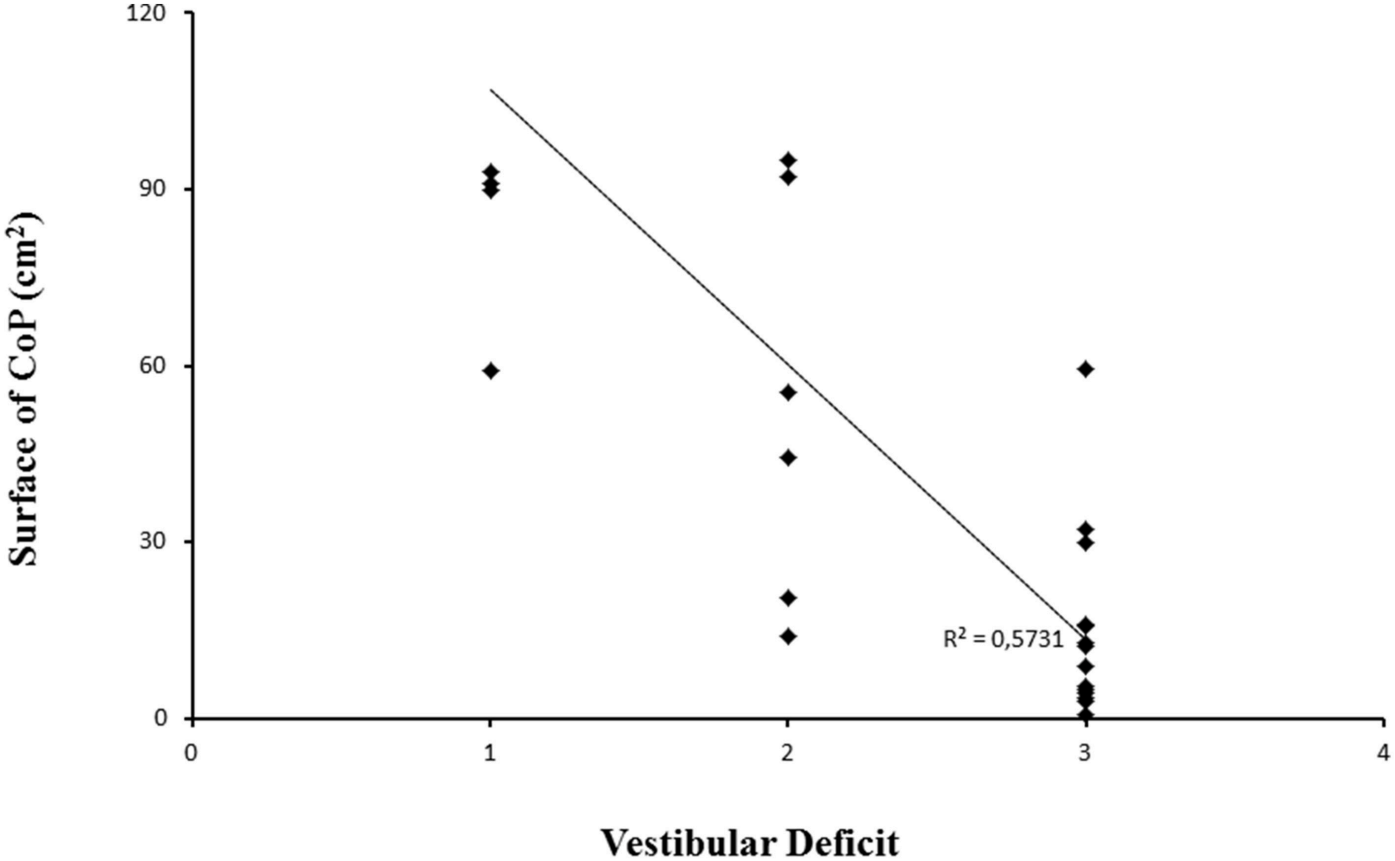
Surface area of CoP for each patient examined with different vestibular function; level 1: complete vestibular areflexia (CVA), level 2: a partial vestibular deficit (PVD), and level 3 means a normal vestibular function (NVF). Line represents the corresponding regression.

We explored further the impact of vestibular deficits, as well as visual deficits on the surface area of CoP on unstable platform with EO condition (Figure 2), and we found a negative significant correlation only between vestibular function and surface area of CoP (*r* = −0.76; *p* < 0.05). We chose to test this correlation only during unstable platform with EO condition because it was the only condition that allowed us to distinguish patients with Usher type I from patients with Usher type II.

### Mean Velocity of CoP

**Figure 3:**
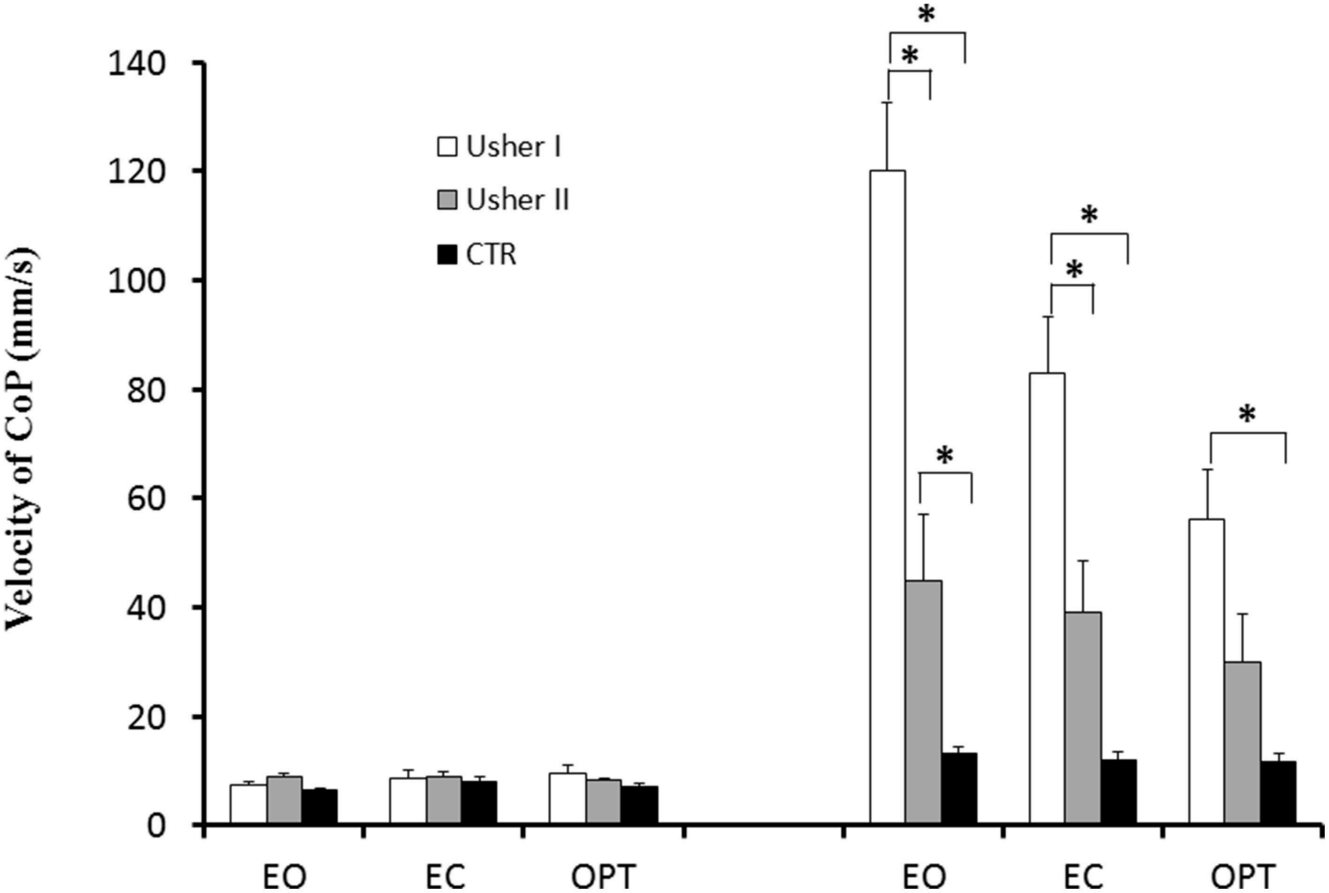
Means and standard deviations of mean velocity of the CoP (mm/s) in two different postural conditions (standing on stable and unstable platform) for the three visual conditions tested in patients with Usher type I and type II and in controls. Asterisks indicate significant difference between the groups (ANCOVA-Bonferroni).

The mean velocity of the CoP for the three groups of subjects recorded under the different postural and visual conditions is shown in Figure 3. ANCOVA showed a significant group effect [*F*_(2, 38)_ = 19.18; *p* < 0.0001]. Bonferroni test showed that patients with Usher type I had higher mean velocity of CoP when compared to CTR and patients with Usher type II (respectively, *p* < 0.004 and *p* < 0.0001), and patients with Usher type II had higher mean velocity of CoP with respect to CTR (*p* < 0.02). A postural condition effect was also found [*F*_(1, 38)_ = 7.23; *p* < 0.011]; mean velocity of CoP on the unstable platform was significantly higher with respect to those measured on stable platform (*p* < 0.0001). A vision condition effect was also found [*F*_(2, 76)_ = 3.69; *p* < 0.030], and Bonferroni test showed that all participants had higher mean velocity of CoP during EO and EC conditions with respect to OPT (respectively, *p* < 0.0001 and 0.01).

We found also a significant interaction between Posture and Group [*F*_(2, 38)_ = 19.01; *p* < 0.0001]. Bonferroni test showed that, on the unstable platform, patients with Usher type I had higher mean velocity of CoP compared to CTR and Usher type II groups (both *p* < 0.0001). Moreover, Usher type II had higher mean velocity of CoP with respect to CTR group (*p* < 0.003).

We found also a significant interaction between Vision and Group [*F*_(4, 76)_ = 6.60; *p* < 0.0001]. Bonferroni test showed that only in EO and EC visual conditions, patients with Usher type I had a higher mean velocity of CoP when compared to CTR (both *p* < 0.0001), and only in EO condition with respect to Usher type II group (*p* < 0.0001). Moreover, there was not any difference between Usher type II and CTR.

Finally there was also a significant interaction between Groups, Vision and Posture [*F*_(4, 76)_ = 7.25; *p* < 0.0001]. Bonferroni test showed that, on the unstable platform, patients with Usher type I had higher mean velocity of CoP in all visual conditions when compared to CTR (respectively, *p* < 0.0001, *p* < 0.0001, and *p* < 0.05), and only in EO and EC visual conditions when compared to patients with Usher type II (respectively, *p* < 0.0001 and *p* < 0.006). Furthermore, patients with Usher type II had higher mean velocity of CoP only in EO visual condition when compared to CTR (*p* < 0.02).

### Postural Instability Index (PII)

**Figure 4:**
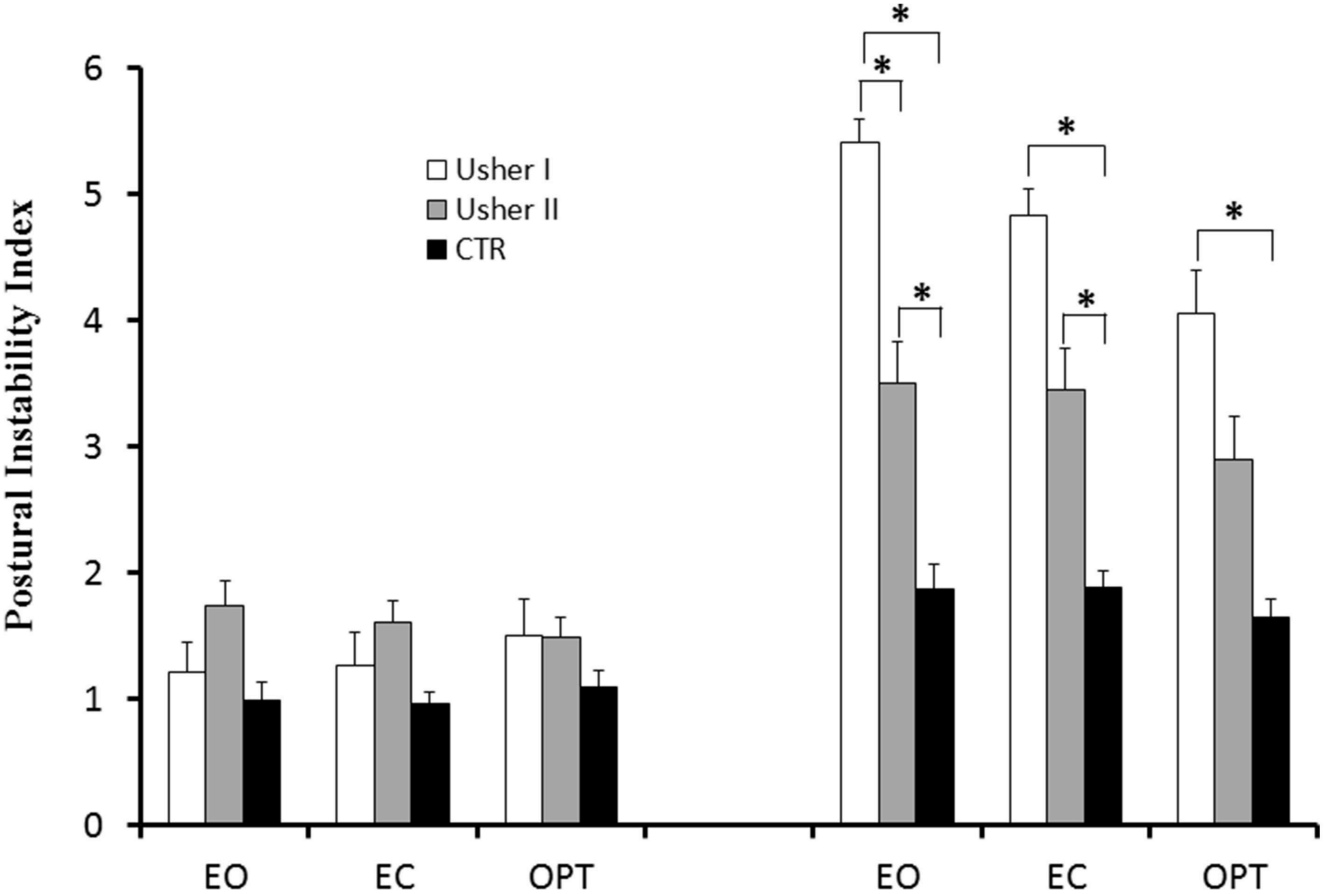
Means and standard deviations of postural instability index (PII) in two different postural conditions (standing on stable and unstable platform) for the three visual conditions tested in patients with Usher type I and type II and in controls. Asterisks indicate significant difference between the groups (ANCOVA-Bonferroni).

The PII for the three groups of subjects recorded under the different postural and visual conditions is shown in Figure 4; ANCOVA reported a significant group effect [*F*_(2, 38)_ = 23.56; *p* < 0.0001]. Bonferroni test showed that all patients with Usher (type I and II) had higher PII when compared to CTR (both *p* < 0.0001). A postural condition effect was found [*F*_(1, 38)_ = 17.07; *p* < 0.0001]: the PII was higher on the unstable platform. We found a significant interaction between Posture and Group [*F*_(2, 38)_ = 27.97; *p* < 0.0001]. Bonferroni test reported that, on the unstable platform, all patients (with Usher type I and II) had a higher PII when compared to CTR (all *p* < 0.0001). Moreover, Usher Type I had a higher PII with respect to Usher type II group (*p* < 0.001). Finally there was also a significant interaction between Group, Vision and Posture [*F*_(4, 76)_ = 3.30; *p* < 0.015]. Bonferroni test reported that, on the unstable platform, patients with Usher type I had higher PII value in all visual conditions when compared to CTR (all *p* < 0.0001), and only during EO condition when they were compared to patients with Usher type II (*p* < 0.01). Furthermore, patients with Usher type II had higher PII only in EO and EC visual conditions when compared to CTR (respectively, *p* < 0.021 and *p* < 0.003).

Finally, the ANOVA on DHI score showed a significant group effect [*F*_(1, 23)_ = 9.53; *p* < 0.005]. Patients with Usher type I had higher scores of DHI with respect to Usher type II; this means that the handicap of patients with Usher type I was increased by their vestibular deficit, affecting their daily life more than Usher type II.

### Effect of Cochlear Implantation on Postural Stability

We did not observe any significant difference on postural stability between the conditions implant on and implant off on stable and unstable platforms for all five patients.

## Discussion

The main findings here are: (i) on the stable platform, postural stability was not significantly different between Usher type I or II patients or controls; (ii) on the unstable platform, with eyes open, the CoP surface of patients with Usher type I was greater than patients with Usher type II and controls, while Usher type II patients were more unstable than controls for mean velocity and PII parameters; (iii) the CoP surface on the unstable platform was significantly different between Usher type I (with partial or complete vestibular loss) and type II (with normal vestibular function); (iv) some Usher type I patients (6/11) had residual vestibular functions, and there was a significant correlation between the level of vestibular function and the CoP surface on the unstable platform with eyes open for all patients; (v) Usher type I patients had significantly higher DHI scores than Usher type II patients.

We showed that visual and vestibular impairment can have a severe impact on postural stability in Usher syndrome. Note, however, that on the stable platform Usher patients' stability measures were not different from those of the control group, even if visual inputs were absent or perturbed. Research has suggested that vestibular loss patients achieve postural control only by means of ankle proprioceptive and plantar somatosensory cues (31). This means that on stable surfaces, Usher patients can reweight visual, proprioceptive, and (when present) vestibular integration in order to maintain good postural stability.

Indeed, reports indicate that sensory inputs such as visual, proprioceptive, and vestibular cues are essential to maintaining normal postural control (32). Several studies have shown that postural control changes in tests with subjects who have impaired vision (12, 33, 34), or who have vestibular deficits (22, 35). In our study, we included patients who only have visual deficits (Usher type II), and patients who have both visual and vestibular deficits (Usher type I). Evaluating postural control on the unstable platform allows us to modify proprioceptive information and to test other systems' (vision and vestibular inputs) contribution to postural stability, as EC and OPT (suppress or perturb visual information). Our results showed that patients with Usher (type I or II) were not able to control their postural stability when the unstable platform disrupted proprioceptive inputs, in comparison to CTR. In similar conditions, healthy adult subjects were able to reweight their sensory systems in order to obtain normal body stability, as Assländer and Peterka (13) show. Most likely due to their visual and/or vestibular deficits, Usher patients were not able to use adaptive mechanisms to control their postural stability on unstable ground.

With open eyes on the unstable platform, only patients with Usher type I were more unstable than Usher type II patients, who were indistinguishable from the control group. Indeed, Usher type I patients have poor vestibular capabilities and reduced visual inputs. The visual deficits (related to RP) start earlier in Usher type I patients (in the first decade of life) than in Usher type II patients (RP starts during the second or third decade of life). Studies have shown that the visual system is necessary for effective integration of sensory systems in early development (36). Consequently, the early visual deficits in Usher type I patients may have a greater impact on multisensory integration, leading to worse postural stability than in patients with Usher type II. In other words, on the unstable platform with EO, Usher type II patients can still use visual and vestibular information to maintain their postural stability in ways not dissimilar from those of controls. However, Usher type I patients on the unstable platform have to rely on visual information only (since the visual system has endured partial impairment from a younger age), as vestibular information is absent or severely impaired.

Usher type II patients are more unstable than controls only in open-eye conditions for mean velocity and in both open- and closed-eye conditions for the PII. These findings underline the importance of visual and vestibular inputs in maintaining posture stability, which the studies from Bucci's group confirm (see section Introduction). We might wonder about the importance of visual inputs for healthy subjects, given that we know postural capability is better in EO than in EC conditions. Indeed, inspecting our data, we could see that, at least for healthy subjects, postural parameters still show smaller values in EO conditions than in EC conditions, even if such a difference is tiny. We suggest that attention capabilities could play a major role in controlling posture, leading to a smaller difference between EO and EC conditions (see also the other papers from Bucci's group). We observed that only the parameter of the surface area of CoP could differentiate between behaviors of patients with Usher type I and patients with Usher type II. Recall that the surface quantifies spatial variability of CoP; it would be interesting to analyze this parameter on unstable platforms only for future studies on Usher syndrome. Several studies reported that the surface of CoP made distinguishing between various pathological groups of subjects, for instance patients with AMD (37) and with strabismus (10), easier in comparison with control subjects. In our Usher patients, the surface of CoP changes significantly between patients with Usher type I and patients with Usher type II. A significant correlation between vestibular functions and body stability also corroborates this finding. This parameter on unstable platform merits further study in Usher patients but also in vestibular deficient patients.

Finally, several studies in the literature have analyzed the impact of postural training on brain connectivity with structural changes. Studies also report a reduction of gray and white matter volume in frontal and motor areas in professional female ballet dancers (38). They also show that sporting activities could modify brain connectivity or plasticity in patients with brain injury or neurodegenerative disorders (39). Hummel et al. (40) studied white matter diffusion ability in subjects with bilateral vestibular failure, balance-trained subjects, and controls using diffusion tensor imaging. They found that both groups of subjects showed similar reduction of white matter diffusion in comparison with control subjects. These authors suggested that, in both subjects with bilateral vestibular failure and balance-trained subjects, brain connectivity changed as subjects exerted similar effort to reach their respective postural stability. Specific balance training can help improve imbalance in people with a lack of vestibular function by changing the brain connectivity, particularly by increasing the radial diffusivity component in the white matter (40). In the future, studies combining postural measures and specific vestibular and visual rehabilitative therapy for Usher patients will be necessary to assess the progress and improvement of these patients objectively. Moreover, studies could investigate treatment approaches using invasive- or non-invasive vestibular prosthetics for potential use in such patients to improve their quality of life (41).

Finally, the quality of life as the DHI evaluates it shows a greater vestibular deficit impact on Usher type I patients than on type II patients, who have normal vestibular function and similar visual and auditory deficits. However, further studies are necessary to evaluate the impact of this multisensorial deficit on the quality of life, as well as to assess how to improve it.

## Limitations and Strengths

Further studies with a larger number of subjects with Usher syndrome will be necessary in order to evaluate the impact of cochlear implants on postural parameters. However, we were able to recruit quite a large number of subjects, which allowed us to investigate the impact of vestibular and visual function on postural control in two groups of patients with Usher types I and II.

## Conclusion

In the present study, we showed the impact on postural stability of visual and vestibular impairment in patients with Usher syndrome. We showed that patients with Usher type I could have some residual vestibular function and that postural measurement (surface of the center of pressure) can differentiate the postural handicap of patients with Usher type I from complete or partial vestibular impairment of patients with Usher type II. In the future, studies could use the surface of the center of pressure to evaluate the effect of postural reeducation and rehabilitation for these patients, as well as the rate of improvement in their quality of life.

## Data Availability

The datasets generated for this study are available on request to the corresponding author.

## Author Contributions

MB and SW-V: conceptualization. IA: selection of patients. SC, MT, and MB: postural measure and data analysis. SC, MT, MB, and SW-V: writing original draft. IA and TA: review and editing.

### Conflict of Interest Statement

The authors declare that the research was conducted in the absence of any commercial or financial relationships that could be construed as a potential conflict of interest.
